# Electrocardiographic Characteristics and Their Correlation with Echocardiographic Alterations in Fabry Disease

**DOI:** 10.3390/jcdd9010011

**Published:** 2022-01-03

**Authors:** Matthew Zada, Queenie Lo, Siddharth J. Trivedi, Mehmet Harapoz, Anita C. Boyd, Kerry Devine, Norman Sadick, Michel C. Tchan, Liza Thomas

**Affiliations:** 1 Westmead Clinical School, Westmead Hospital, University of Sydney, Sydney 2145, Australia; matthew.zada@sydney.edu.au (M.Z.); siddharth.trivedi22@gmail.com (S.J.T.); mehmet.h11@gmail.com (M.H.); normansadick@gmail.com (N.S.); 2 Department of Cardiology, Westmead Hospital, Sydney 2145, Australia; 3 South Western Sydney Clinical School, Liverpool Hospital, University of New South Wales, Sydney 1466, Australia; queenlo@hotmail.com; 4 Westmead Private Cardiology, Sydney 2145, Australia; boydac@iinet.net.au; 5 Genetic Medicine, Westmead Hospital, Sydney 2145, Australia; Kerry.Devine@health.nsw.gov.au (K.D.); Michel.Tchan@health.nsw.gov.au (M.C.T.)

**Keywords:** Fabry disease, electrocardiogram, transthoracic echocardiography, LV hypertrophy, global longitudinal strain, basal longitudinal strain

## Abstract

Fabry disease (FD) is an X-linked disorder with α-galactosidase A deficiency. Males (>30 years) and females (>40 years) often present with cardiac manifestations, predominantly left ventricular hypertrophy (LVH). The aim of this study was to evaluate electrocardiographic (ECG) characteristics within FD patients to identify gender related differences, and to additionally explore the association of ECG parameters with structural and functional alterations on transthoracic echocardiography (TTE). Retrospective cross-sectional analysis of 45 FD patients with contemporaneous ECG and TTE was performed and compared to age and gender matched healthy controls. FD patients demonstrated alterations in several ECG parameters particularly in males, including prolonged P-wave duration (91 vs. 81 ms, *p* = 0.022), prolonged QRS duration (96 vs. 84 ms, *p* < 0.001), increased R-wave amplitude in lead I (8.1 vs. 5.7 mV, *p* = 0.047), increased Sokolow–Lyon index (25 vs. 19 mV, *p* = 0.002) and were more likely to meet LVH criteria (31% vs. 7%, *p* = 0.006). FD patients with impaired basal longitudinal strain (LS) on TTE were more likely to meet LVH criteria (41% vs. 0%, *p* = 0.018). Those with more advanced FD (increased LV wall thickness on TTE) were more likely to meet LVH criteria but additionally demonstrated prolonged ventricular depolarization (QRS duration 101 vs. 88 ms, *p* = 0.044). Therefore, alterations on ECG demonstrating delayed atrial activation, delayed ventricular depolarization and evidence of LVH were more often seen in male FD patients. Impaired basal LS, a TTE marker of early cardiac involvement, correlated with ECG abnormalities. Increased LV wall thickness on TTE, a marker of more advanced FD, was associated with more severe ECG abnormalities.

## 1. Introduction

Fabry disease (FD) is an X-linked lysosomal storage disorder due to α-galactosidase A enzyme deficiency, with resultant progressive accumulation of globotriaosylceramide (Gb3) in various organ tissues [1]. There are a variety of clinical manifestations including neurological, gastrointestinal, renal and cardiac in affected individuals [2]. Whilst many symptoms occur in childhood, cardiac symptoms usually do not present until the third or fourth decade [3]. 

FD patients with cardiac involvement develop several manifestations, typically with progressive increase in left ventricular (LV) wall thickness (on transthoracic echocardiography (TTE) or on cardiac magnetic resonance imaging (CMR)), significant life-threatening arrythmias or conduction defects, interstitial remodeling and myocardial fibrosis (as demonstrated on myocardial biopsy) [4]. Electrocardiographic (ECG) alterations have been reported including altered conduction, demonstrated by a prolonged PQ interval and QRS duration, as well as ECG features of LVH [5,6,7]. Prior studies of FD patients have shown a correlation between prolonged QRS duration on ECG and LV mass on TTE [8] and CMR [9]. Early diagnosis of FD is imperative as the evidence suggests that LVH may be prevented or may regress with early treatment with enzyme replacement therapy (ERT); moreover, in advanced FD, the response to ERT is poor [10]. Recently, there has been interest in identifying ECG parameters associated with cardiac involvement prior to the development of LVH [11,12]. 

As FD is an X-linked condition, traditionally female heterozygotes were considered carriers [13]; however, it is now widely accepted that female FD patients can develop cardiomyopathy, but with delayed onset and slower progression [14]. Variable findings have been reported in relation to ECG changes in females with FD [6]. 

In this retrospective cross-sectional study, our aim was to characterize ECG parameters in FD patients compared to healthy controls and evaluate gender related differences in ECG parameters. We further sought to examine the relationship between two widely available investigations and evaluate the correlation between ECG parameters with structural (e.g., LV mass) and functional changes (e.g., longitudinal strain) on TTE. In particular, we examined the relationship of ECG parameters with basal longitudinal strain, a marker of early cardiac involvement [15].

## 2. Materials and Methods

We retrospectively analyzed data from 45 genetically confirmed FD patients (mean age 42 yrs, 26/45 males) from the Department of Genetic Medicine at Westmead Hospital between 2009–2014 (baseline characteristics in Supplementary Table S2). The majority (38) of patients had contemporaneous TTE and ECG performed prior to ERT therapy; whilst only 7 patients had already been commenced on ERT at the time of their TTE and ECG. Subsequently, a further 19 FD patients received ERT based on current guidelines in Australia. FD patients were compared to age and gender matched healthy controls from a departmental database, with no documented cardiovascular disease or risk factors and were not on any medications. Ethical approval was obtained from the South West Sydney Health District Human Research Ethics Committee.

### 2.1. Electrocardiography 

**Figure 1:**
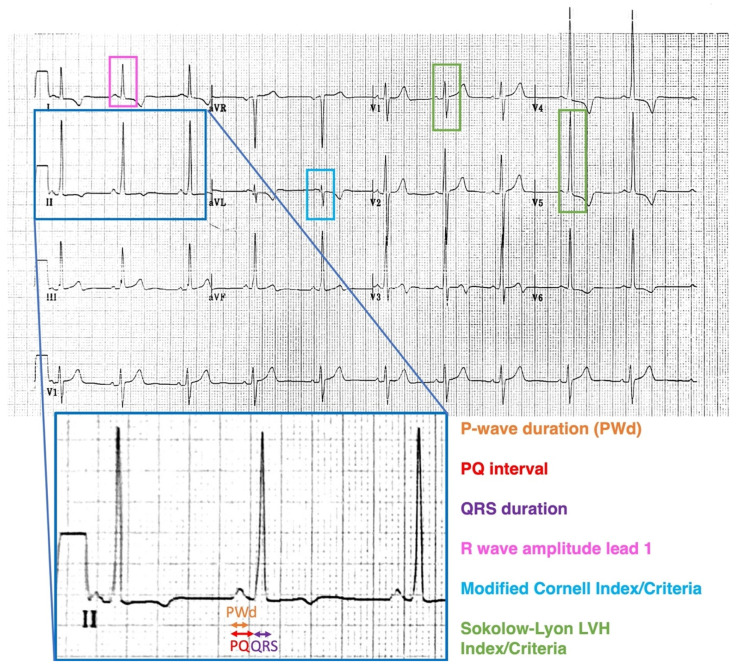
ECG parameters assessed included PQ interval in lead II and lead V1, P wave duration (PWd) in lead II and V1, QRS duration in lead II, R wave amplitude in lead 1, Sokolow–Lyon index and criteria, Modified Cornell index and criteria and complete right bundle branch block criteria.

Standard 12-lead ECGs were performed, recorded at 50 mm/s with an amplitude of 1 mV/10 mm. An average of 3 measurements were performed manually with the aid of calipers for all parameters. Figure 1 demonstrates the ECG parameters that were assessed including PQ interval (average of PQ interval in lead II and V1), P-wave duration (average of P-wave duration in lead II and V1), QRS duration (in lead II), R wave amplitude in lead I, Sokolow–Lyon index (sum of voltage of S wave in V1/V2 and R wave in V5/V6) using the previously described criteria and the Modified Cornell Index (measuring R wave amplitude in aVL with patients meeting LVH criteria if >12 mm) [16]. PQ interval and P-wave duration were corrected to a heart rate of 60 by dividing intervals by the heart rate and multiplying by 60. ECGs were assessed for meeting LVH (Sokolow–Lyon criteria) and right bundle branch block (RBBB). ECG parameters were compared between the following groups: FD patients vs. controls (Table 1A); male FD patients vs. male controls (Table 1A); female FD patients vs. female controls (Table 1A); and male FD patients vs. female FD patients (Table 1B). We examined ECG findings in subgroups of FD patients based on TTE criteria: increased LV wall thickness vs. normal LV wall thickness, impaired GLS vs. normal GLS and impaired basal longitudinal strain (LS) vs. normal basal LS (Supplementary Table S1). 

### 2.2. Echocardiography

A comprehensive TTE was performed using commercially available ultrasound systems (Vivid 7 or E9 ultrasound scanner; GE Healthcare, Horten, Norway) with a 2.5-MHz transducer; M-mode, 2D, color, Doppler and tissue Doppler imaging were obtained in accordance with American Society of Echocardiography (ASE) guidelines [17]. Digital images were stored for offline analysis. One patient was excluded from analysis due to poor image quality. An average of 3 measurements were used for all echocardiographic parameters. LV wall thickness was measured from 2D images from the parasternal long axis view in end diastole; FD patients were stratified based on ASE guidelines to have increased wall thickness if ≥11 mm for males and ≥10 mm for females [18]. LV mass was calculated using the Devereux method as per ASE criteria and indexed (LVMI) to body surface area (BSA) [19]; gender-based cutoffs for increased LV mass used were >115 g/m^2^ for males and >95 g/m^2^ for females. LVEF was calculated using Simpson’s biplane method by measuring LV end diastolic and end systolic volumes from apical−4 and −2 chamber views. Biplane maximum left atrial volume at end systole was measured from the apical−4 and −2 chamber views and indexed to body surface area (LAVI). Peak E and peak A velocities were obtained from pulsed Doppler mitral inflow. Tissue Doppler velocities (e’) were measured from the septal and lateral mitral annulus. The ratio of E velocity over average of septal and lateral e’ velocity was determined (E/e’). (Table 2).

### 2.3. Strain Analysis

2D global longitudinal strain (GLS) analysis was performed using customized offline computer software (EchoPAC, GE, Horten, Norway, Version 201), from zoomed apical LV 4-, 2- and 3-chamber views, obtained at high frame rates (>55 fps). The endocardial border was manually traced in end-systole and the width of the region of interest (ROI) was manually adjusted to track the myocardium. Strain from up to 2 segments could be excluded in one imaging plane; if >2 segments could not be adequately tracked, the strain measurement from this plane was omitted. Impaired GLS was defined as GLS worse than −18.0% [20] and impaired basal longitudinal strain (LS) was defined as basal LS worse than −18.1% [15].

### 2.4. Inter-Observer and Intra-Observer Variability

To assess intra-observer variability, TTE and ECG of 10 randomly selected patients were reviewed by the same investigator 4 weeks apart. Inter-observer variability was assessed by a second blinded investigator in the same 10 patients.

### 2.5. Statistical Analysis

Continuous variables are expressed as a mean, and categorical variables as a percentage. Non-parametric tests (Mann-Whitney) were performed to examine differences in means between FD patients and controls and between male and female FD patients. Chi-square analysis was performed to examine differences in categorical variables. Pearsons correlation was performed to evaluate associations between continuous variables. Statistical analyses were performed using Statistical Package for Social Sciences, version 22 (SPSS, Chicago, IL, USA) and MedCalc version 15.8 (MedCalc Software Ltd., Ostend, Belgium) with *p* < 0.05 considered statistically significant. Inter and intra-observer variability were evaluated using intra-class correlation (ICC) and the coefficient of variation (CoV).

## 3. Results

### 3.1. ECG in FD versus Controls

All FD patients were in sinus rhythm. Analysis by gender (Table 1A) showed that corrected PQ interval was longer in male FD patients (169 vs. 148 ms, *p* = 0.027) but not so for female FD patients (126 vs. 121 ms, *p* = 0.729). The corrected P-wave duration was longer in FD patients (91 vs. 81 ms, *p* < 0.022), but similar to the corrected PQ interval, was significantly longer for male FD patients (105 vs. 88 ms, *p* = 0.008), but not so in female FD patients (74 vs. 72 ms, *p* = 0.644). The QRS duration was significantly longer in FD patients compared to controls (96 vs. 84 ms, *p* < 0.001) and again, QRS duration was significantly longer in male FD patients (103 vs. 85 ms, *p* < 0.001), but not so in female FD patients (87 vs. 82 ms, *p* = 0.271). 

Amplitude of the QRS complex was higher in FD patients in lead I (8.1 vs. 5.7 mV, *p* = 0.047) and in the chest leads (25 vs. 19 mV, *p* < 0.002). When analyzed by gender, QRS amplitude was higher in male FD patients both in lead I (9.1 vs. 5.8 mV, *p*= 0.014) and the chest leads (28.4 vs. 19.6 mV, *p* = 0.008), but not for female FD patients (lead I: 6.7 vs. 5.1 mV, *p* = 0.885; chest leads: 21.5 vs. 17.7 mV, *p* = 0.065). FD patients were more likely to meet Sokolow–Lyon criteria for LVH (31% vs. 7%, *p* = 0.006) and male FD patients met LVH criteria more frequently versus controls (42% vs. 8%, *p* = 0.009), though not observed in female FD patients (16% vs. 5%, *p* = 0.604). There was no significant difference in the presence of RBBB in FD patients compared to controls (11% vs. 4%, *p* = 0.434). 

### 3.2. Male FD vs. Female FD: ECG and Echocardiographic Correlates

The corrected PQ interval, P wave duration and QRS duration were significantly longer in male FD patients compared to female FD patients (Table 1B). The average voltage was higher in male FD patients compared to female FD patients in the chest leads (28.4 vs. 21.5 mV, *p* = 0.043) but not in lead I (9.1 vs. 6.7 mV, *p* = 0.051). Male FD patients met LVH criteria more often than females, though not statistically significant (42% vs. 16%, *p* = 0.102). Only male FD patients had RBBB when compared to females (5/26 vs. 0/19). Male FD patients were more likely to have increased LV wall thickness on TTE (77% vs. 37%, *p* = 0.013). Strain analysis demonstrated that male and female FD patients were as likely to have impaired GLS (69% vs. 55%, *p* = 0.525) and impaired basal LS (89% vs. 61%, *p* = 0.064).

### 3.3. Correlation of Electrocardiographic and Echocardiographic Markers 

**Figure 2:**
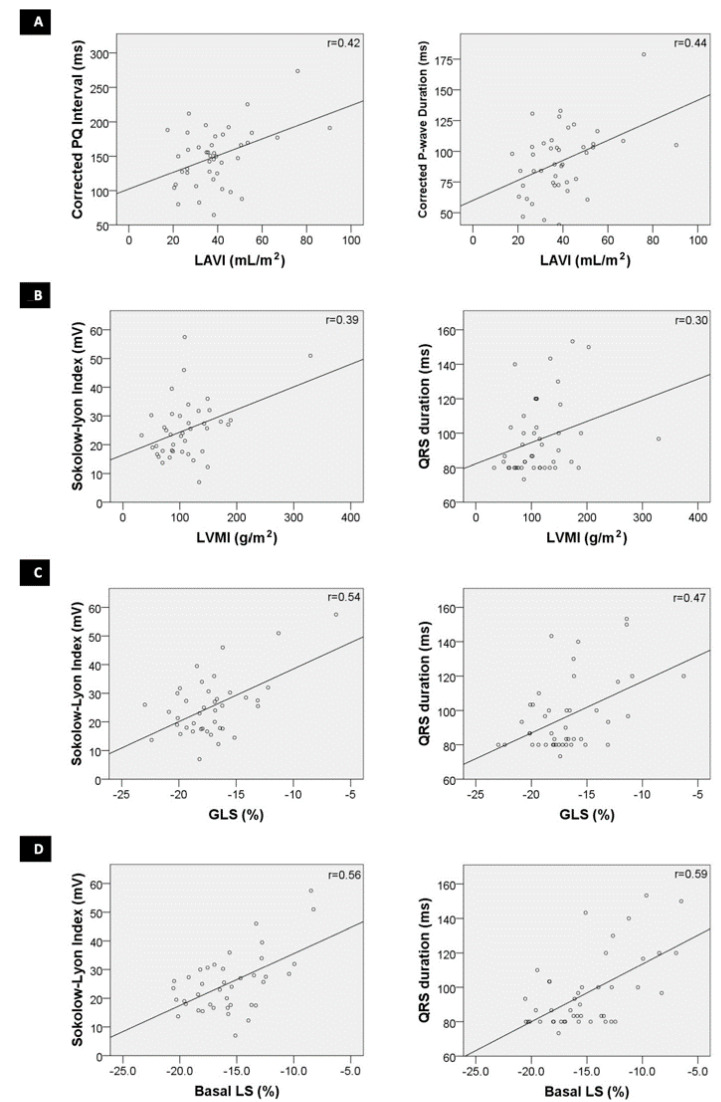
Correlation between electrocardiographic and echocardiographic parameters; (**A**)—Correlation between corrected PQ interval and maximum left atrial volume indexed to body surface area (LAVI) and between corrected P-wave duration and LAVI; (**B**)—Correlation between Sokolow–Lyon index and left ventricular mass indexed to body surface area (LVMI) and between QRS duration and LVMI; (**C**)—Correlation between Sokolow–Lyon index and Global Longitudinal Strain (GLS) and between QRS duration and GLS; and (**D**)—correlation between Sokolow–Lyon index and Basal Longitudinal Strain (LS) and between QRS duration and Basal LS.

There was a significant correlation (Figure 2) between LAVI and the corrected PQ interval (*r =* 0.42, *p* = 0.005) as well as the corrected P-wave duration (*r =* 0.48, *p* = 0.003). 

There was a significant correlation (Figure 2) between LVMI on TTE with QRS duration (*r =* 0.30, *p* = 0.044) and with Sokolow–Lyon index on ECG (*r =* 0.39, *p* = 0.011). Similarly, there were significant correlations between GLS and QRS duration (*r =* 0.47, *p* = 0.001) and Sokolow–Lyon index (*r =* 0.54, *p* < 0.001), as also between basal LS and QRS duration (*r =* 0.59, *p* < 0.001) and Sokolow–Lyon index (*r =* 0.56, *p* = 0.004). 

### 3.4. ECG Characteristics in FD Patients with Altered TTE Parameters: Increased Wall Thickness, Altered GLS and Basal Segmental LS

Subgroup analysis of FD patients (Supplementary Table S1) showed that those with increased wall thickness on TTE (*n* = 27) had a significantly longer QRS duration (101 vs. 88 ms, *p* = 0.044) and higher QRS complex amplitude in lead I (9.9 vs. 5.4 mV, *p* = 0.002) but not so in the chest leads (27.7 vs. 21.8 mV, *p* = 0.112). As expected, FD patients with increased wall thickness on TTE were also more likely to meet LVH criteria on ECG (48% vs. 6%, *p* = 0.003). 

FD patients with impaired GLS (*n* = 28) had higher QRS complex amplitude in lead I (9.7 vs. 5.7 mV, *p* = 0.008) and were more likely to meet LVH criteria on ECG (43% vs. 13%, *p* = 0.049). Similarly, FD patients with impaired basal LS (*n* = 34) had higher QRS complex amplitude in lead I (9.1 vs. 5.1 mV, *p* = 0.014) and were also more likely to meet LVH criteria on ECG (41% vs. 0%, *p* = 0.018). 

We examined ‘stages’ of disease progression by dividing the FD patients into three groups: (1) EARLY (Group A): normal LV wall thickness and normal basal segmental LS; (2) INTERMEDIATE (Group B): normal LV wall thickness and impaired basal segmental LS; and (3) LATE (Group C): increased LV wall thickness and impaired basal LS. There were significant differences in ECG and TTE parameters (Table 2), albeit acknowledging the relatively small numbers in groups 1 and 2. Two female FD patients with hypertension and mildly increased LV wall thickness (likely due to hypertension) with normal basal LS were excluded from this subgroup analysis.

### 3.5. Reproducibility of ECG Analysis

The ICC for inter-observer variability for the PQ interval was 0.90 (95% confidence interval [CI], 0.62–0.98; coefficient of variation (COV), 6.22%) whilst the intraobserver variability for the PQ interval was 0.92 (95% CI, 0.68–0.98; COV = 5.47%). The ICC for inter-observer variability for the QRS duration was 0.86 (95% CI, 0.45–0.97; COV = 6.32%) whilst the intraobserver variability for the QRS duration was 0.84 (95% CI 0.35–0.96; COV = 5.65%). The ICC for inter-observer variability for LVH Sokolow–Lyon index was 0.97 (95% CI 0.89–0.99; COV = 7.87%) whilst the intraobserver variability for LVH Sokolow–Lyon index was 0.99 (95% CI 0.95–0.99; COV = 5.31%). 

We have previously reported inter- and intra-observer variability for TTE parameters GLS and segmental strain [15].

## 4. Discussion

In this single center cohort of FD patients, males more commonly had alteration in ECG parameters including prolonged corrected PQ interval (i.e., lengthened P-wave duration), prolonged QRS duration and evidence of LVH on ECG. These ECG alterations were not observed as frequently in female FD patients. Prolonged QRS duration and Sokolow–Lyon index correlated with traditional TTE abnormalities (increased LV wall thickness) but had a stronger correlation with novel TTE markers such as GLS and basal LS. 

### 4.1. ECG Parameters in FD Males and Females

Our results support previous observations that significant ECG alterations are more likely to be present in FD males. Despite one study not finding significant differences in the PQ interval and QRS interval between FD males and females [6], the study of Niemann et al. evaluating 150 FD patients (63 males and 87 females; average age of 38 years), demonstrated that FD males were more likely to have prolonged QRS duration, higher Sokolow–Lyon index and RBBB criteria [9]. Additionally, we identified altered PQ interval, P wave duration and QRS duration in FD males. In general, in FD, cardiac involvement occurs earlier and more commonly in males [14]. Therefore, our observations suggest that FD males, albeit at similar or younger age than female FD patients were at a later stage in the disease course, with more males having increased wall thickness on TTE and more ECG alterations.

### 4.2. Alterations in Conduction: PQ Interval and P-Wave Duration

Interestingly, young patients with early-stage FD have been reported to have PQ interval shortening, as a result of P wave shortening [5,6], without evidence of an accessory pathway [21]; however, none of the patients in our study demonstrated this. Potential mechanisms for enhanced conduction velocity include accumulation of Gb3 in cardiomyocytes, increase in diameter of conducting cells and direct interaction between Gb3 and ion channels involved in action potential propagation [5,6]. In other glycogen storage disorders, it is thought that electrophysiological phenomena such as PQ interval shortening are directly related to glycogen storage in and around the atrioventricular node [22]. ERT normalizes the shortened PQ intervals and shortened P waves [23]. As FD progresses, the extent of infiltration worsens with macroscopic changes to the myocardium, causing PQ interval prolongation (corresponding to left atrial enlargement) [5]. This theory is supported by the correlation of LAVI to PQ interval in our cohort.

### 4.3. Alterations in Conduction: QRS Interval and Right Bundle Branch Block

Patients in early-stage FD have QRS interval shortening, likely a result of enhanced conduction velocity [5,6]. With disease progression, the QRS interval prolongs [7]. In patients with LVH (from any cause), prolonged QRS interval is often seen and is thought to be due to the increased time taken for left ventricular depolarization [24,25,26]. As previously shown by Kampmann et al. [8], our cohort also demonstrated prolonged QRS duration on ECG, likely secondary to increased LV mass, considering almost two-thirds of our cohort demonstrated increased LV wall thickness on TTE (27/45). 

Similar to previous studies, our cohort demonstrated right bundle branch block more commonly than left bundle branch block [9,27,28]. We hypothesize that accumulation of Gb3 occurs more often within the right bundle than within the left bundle in patients with FD. There is certainly histological evidence reported in the literature of vacuolization within myocytes with preferential extension into the right bundle [29].

### 4.4. ECG for Detecting LVH (Sokolow–Lyon Criteria) 

With worsening cardiomyopathy in FD, LV wall thickness increases with an increased Sokolow–Lyon index on ECG [7]. FD males in our study showed evidence of LV hypertrophy with several meeting the Sokolow–Lyon criteria on ECG. This is largely attributable to male FD patients being at ‘a later stage’ of their disease, evidenced by 20/26 male FD patients having increased LV wall thickness on TTE. 

Although 27/45 patients had increased LV wall thickness on TTE, only 13/27 (48%) met Sokolow–Lyon criteria for LVH on ECG. This is in keeping with previous studies that have shown that the sensitivity of ECG at detecting LVH varies depending on the method used but ranges from 20–55%, with specificity decreasing from 95% to 77% as sensitivity increases [25,26,30]. Therefore, even though ECG is a simple method of screening for LV hypertrophy in FD, it is not as sensitive as TTE. 

### 4.5. Correlating Electrocardiographic and Echocardiographic Parameters

Namdar et al. have reported that PQ interval may be prolonged in FD due to left atrial enlargement [5]. We demonstrated a significant correlation between LAVI and both PQ interval and P-wave duration, supporting the concept that the PQ interval is prolonged due to delayed atrial depolarization secondary to left atrial enlargement.

Kampmann et al. analyzed 166 ECGs and TTEs of hemi- and heterozygous FD patients (44% males; 56% females; average age 35 years) and found a significant correlation between Sokolow–Lyon index and QRS duration with LVMI [8], similar to our findings. GLS identifies subclinical cardiac involvement in a variety of conditions [31]; and in FD, GLS can be impaired even with normal LV wall thickness [15]. Augosto et al. have recently described several phases of FD including a silent ‘accumulation stage’ where LV wall thickness is normal, with evidence of detectable infiltration on CMR with corresponding impairment in GLS on TTE [32]. A previous study that used a binary definition to classify patients into 2 groups—normal versus abnormal ECG (defined as any of the following: prolonged or shortened PQ interval; prolonged QRS duration; the presence of LVH criteria; or t wave inversion), also demonstrated correlation with impaired GLS [33].

We sought to further explore the relationship between ECG parameters and strain parameters. In our cohort of 45 FD patients, 28 (62%) had impaired GLS and 34 (76%) had impaired basal LS; whilst 27 (60%) had increased LV wall thickness on TTE (Supplementary Table S1). This would suggest that strain, in particular basal LS, is more sensitive for detecting cardiac involvement in FD (with strain impaired in FD patients with normal LV wall thickness). FD patients with impaired basal LS demonstrated ECG abnormalities such as Sokolow–Lyon LVH criteria, but not QRS prolongation. Whereas patients with increased LV wall thickness on TTE, represent a group with more advanced disease and more severe ECG abnormalities (Sokolow–Lyon LVH criteria as well as QRS prolongation).

To our knowledge, this is the first instance where ECG abnormalities have been correlated to impaired basal LS in FD patients. This has practical implications for patient screening; if for example there is evidence of LVH on an ECG, but the LV wall thickness is normal on TTE, this may prompt the clinician to investigate for cardiac involvement by assessing basal longitudinal strain. Other investigations including cardiac MRI or myocardial biopsy may be confirmatory.

**Figure 3:**
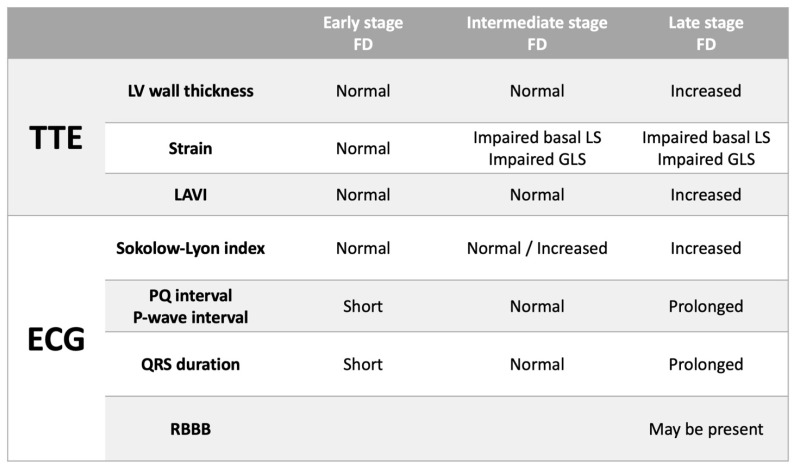
Expected transthoracic echocardiographic (TTE) and electrocardiographic (ECG) changes with progression of Fabry Disease (FD). LAVI = maximum left atrial volume indexed to body surface area. LS = longitudinal strain. GLS = global longitudinal strain. RBBB = right bundle branch criteria.

Therefore, we have proposed a model which highlights these findings and demonstrates corresponding ECG and TTE findings that could be expected with disease progression in FD (Figure 3): In early-stage FD, LV wall thickness, GLS and basal LS are normal—ECG characteristics may include shortened PQ interval and QRS duration due to enhanced conduction (although this was not seen in our cohort probably due to most of our cohort being at a later stage in the disease process); In intermediate stage FD, LV wall thickness is normal although there may be evidence of impairment in longitudinal strain, particularly basal LS—in such patients the PQ interval and QRS duration lengthen so that they lie within the normal range and the Sokolow–Lyon index may be increased; In late-stage FD, LV wall thickness is increased and GLS as well as basal LS are impaired—ECG characteristics would include evidence of LVH (increased Sokolow–Lyon index) and prolongation of the PQ interval and QRS duration (with a predilection for development of RBBB).

### 4.6. Limitations

This study had a modest sample size; however, it is inherently difficult to recruit large numbers of FD patients at a single center, given the low disease prevalence. Despite this we were able to demonstrate differences in ECG characteristics in FD patients.

CMR was not performed at a contemporaneous time as the ECG and TTE. Hence correlation with CMR findings could not be reported meaningfully. 3D echocardiography that allows more accurate estimation of LV mass and cardiac volumes was not performed as several patients had TTE performed prior to 3D technology being widely available.

Hypertensive heart disease may mimic some of the structural changes observed in FD. 60% of FD patients had a history of hypertension; however, all were managed according to guidelines and blood pressure was well controlled except for 1 female FD patient who was hypertensive at the time of assessment (with a systolic blood pressure more than 140 mmHg). Hence it is likely that changes observed are more attributable to FD than coexistent hypertension.

Our study compared FD patients to healthy controls as opposed to those with hypertrophic phenotype (e.g., hypertensive heart disease). However, a previous publication [16] has reported ECG characteristics in varying types of hypertrophic phenotypes.

## 5. Conclusions

ECG abnormalities in FD patients are more common in male FD patients. Alterations in conduction present in FD include prolonged PQ interval, prolonged P-wave duration and prolonged QRS duration (particularly RBBB). Impaired longitudinal strain on TTE is an earlier marker of cardiac involvement in FD and is associated with LVH on ECG. However, increased LV wall thickness on TTE occurs at a later stage in the disease process and in such instances demonstrates LVH and prolonged ventricular depolarization on ECG.

## Figures and Tables

**Table 1:** (**A**). Electrocardiographic characteristics of FD patients compared to age and gender matched normal controls. ECG parameters assessed are listed in the first column. Comparison is made between FD patients and matched controls; between male FD patients and matched male controls only; and between female FD patients and matched female controls only. (**B**) Comparison between the electrocardiographic characteristics of male FD patients and female FD patients. ECG parameters assessed are listed in the first column.

(A)									
	All FD Patients Compared to Matched Controls			Male FD Patients Compared to Matched Male Controls			Female FD Patients Compared to Matched Female Controls		
	FD [*n* = 45]	Controls [*n* = 45]	*p*-Value	Male FD [*n* = 26]	Male Controls [*n* = 26]	*p*-Value	Female FD [*n* = 19]	Female Controls [*n* = 19]	*p*-Value
Age (yrs.)	42	42	0.939	42	42	0.934	42	42	0.84
Corrected PQ Interval (ms)	150	137	0.051	169	148	**0.027 ^†^**	126	121	0.729
Corrected PWD (ms)	91	81	**0.022 ^†^**	105	88	**0.008 ^†^**	74	72	0.644
QRS duration (ms)	96	84	**<0.001 ^†^**	103	85	**<0.001 ^†^**	87	82	0.271
R wave amplitude lead I (mV)	8.1	5.7	**0.047 ^†^**	9.1	5.8	**0.014 ^†^**	6.7	5.6	0.885
Sokolow–Lyon index (mV)	25	19	**0.002 ^†^**	28.4	19.6	**0.008 ^†^**	21.5	17.7	0.065
Sokolow–Lyon LVH criteria	14/45 (31%)	3/45 (7%)	**0.006 ^†^**	11/26 (42%)	2/26 (8%)	**0.009 ^†^**	3/19 (16%)	1/19 (5%)	0.604
Modified Cornell Index (mV)	4.2	3	0.507	5.3	3.2	0.374	2.7	2.7	0.965
Modified Cornell LVH criteria	Apr-45	0/45	0.117	26-Apr	0/26	0.11	0/19	0/19	-
RBBB criteria	5/45 (11%)	2/45 (4%)	0.434	5/26 (19%)	2/26 (8%)	0.419	0/19	0/19	-
(**B**)									
ECG Parameters			Male FD [*n* = 26]			Female FD [*n* = 19]			*p*-value
Age (yrs)			42			42			0.818
Corrected PQ Interval (ms)			169			126			**0.001 ^†^**
Corrected PWD (ms)			105			74			**<0.001 ^†^**
QRS duration (ms)			103			87			**0.004 ^†^**
R wave amplitude lead I (mV)			9.1			6.7			0.051
Sokolow–Lyon index (mV)			28.4			21.5			**0.043 ^†^**
Sokolow–Lyon LVH criteria			11/26 (42%)			3/19 (16%)			0.102
Modified Cornell Index (mV)			5.3			2.7			0.164
Modified Cornell LVH criteria			4/26			0/19			0.126
RBBB criteria			5/26 (19%)			0/19			0.063
LVH on TTE			20/26 (77%)			7/19 (37%)			**0.013 ^†^**
Impaired GLS on TTE			18/26 (69%)			10/18 (55%)			0.525
Impaired Basal LS on TTE			23/26 (89%%)			11/18 (61%)			0.064

Intervals and voltage are expressed as Means. Categorical variables are expressed as frequency and percentage of occurrence in each group. **^†^** *p* < 0.05. PWD = P-wave duration; LVH = Left Ventricular Hypertrophy; RBBB = Right bundle branch block; GLS = global longitudinal strain; LS = longitudinal strain, FD = Fabry disease, ECG = electrocardiograph

**Table 2:** Comparison of clinical, echocardiographic and electrocardiographic markers of FD patients with normal LV wall thickness and normal basal LS; normal LV wall thickness with impaired basal LS; and increased LV wall thickness and impaired basal LS.

	Normal LV Wall Thickness & Normal Basal LS [*n* = 8] [Group A]	Normal LV Wall Thickness & Impaired Basal LS [*n* = 9] [Group B]	Increased LV Wall Thickness & Impaired Basal LS [*n* = 25] [Group C]
**Clinical characteristics**			
Age	31	46 *	45
Males	3 (38%)	3 (33%)	20 (80%)
ERT—no. on ERT at time of simultaneous ECG/TTE	0	1 (11%)	6 (24%)
ERT—total no. who were treated with ERT after ECG/TTE	1 (13%)	4 (44%)	21 (84%)
Albuminuria/Chronic kidney disease	0	1 (11%)	12 (48%)
Dyslipidaemia	1 (13%)	5 (56%)	15 (60%)
Diabetes mellitus	0	2 (22%)	1 (4%)
Arterial hypertension	0	2 (22%)	15 (60%)
Transient ischaemic attack / stroke	1 (13%)	2 (22%)	5 (20%)
Ischaemic heart disease	0	1 (11%)	2 (8%)
**Echocardiographic parameters**			
Average LV wall thickness (mm)	8.3	7.5	14.6 +
Left ventricular mass index (g/m^2^)	73.4	76.9	141.9 +
LVEF (%)	61	60	61
Peak E (cm/s)	98	93	78
Peak A (cm/s)	63	64	66
E/A	1.61	1.63	1.26
Lateral e’ (cm/s)	14	12	9 +
Septal e’ (cm/s)	11	9 *	7
Average E/e’	8.22	9.68	11.0
LAVI (mL/m^2^)	44.6	40.7	36.8
Valvular disease	Moderate MR—1 Mitral prolapse—1 (mild)	Mild MR—2 Mild TR—3	Moderate MR—3 Moderate PR—1 Mild MR—1 Mild TR—2 Mild AS—1
**ECG paramaters**			
Corrected PQ Interval (ms)	138	146	160
Corrected PWD (ms)	85	87	98
QRS duration (ms)	90	88	102
R wave amplitude lead I (mV)	5.1	6.0	10.3+
Sokolow–Lyon index (mV)	20.7	22.6	28.0
Sokolow–Lyon LVH criteria	0/8	1/9 (11%)	13/25 (52%) +
Modified Cornell index (mV)	2.3	2.4	5.7
Modified Cornell LVH criteria	0/8	0/9	4/25 (16%)
RBBB criteria	0/8	1/9 (11%)	4/25 (16%)

Intervals and voltage are expressed as Means. Categorical variables are expressed as frequency and percentage of occurrence in each group. PWD = P-wave duration; LVH = Left Ventricular Hypertrophy; RBBB = Right bundle branch block; GLS = Global longitudinal strain; LS = longitudinal strain; LVEF = Left ventricular ejection fraction; LAVI = Left atrial volume index; MR = mitral regurgitation; PR = pulmonary regurgitation; TR—tricuspid regurgitation; AS = aortic stenosis, FD = Fabry disease, ECG = electrocardiograph. * *p* < 0.05 for Group A vs. Group B. + *p* < 0.05 for Group B vs. Group C.

## Supplementary Materials

The following are available online at https://www.mdpi.com/article/10.3390/jcdd9010011/s1, Table S1: Electrocardiographic characteristics of FD patients in comparison to echocardiographic findings. ECG parameters assessed are listed in the first column. Comparison is made between FD patients with increased LV wall thickness and normal LV wall thickness; FD patients with impaired GLS vs. normal GLS; and between FD with impaired basal LS and normal basal LS, Table S2: Baseline clinical and echocardiographic characteristics of FD patients. Table S3. Comparison of clinical, echocardiographic and electrocardiographic markers of FD patients with normal LV wall thickness (cut off 13mm). and normal basal LS; normal LV wall thickness with impaired basal LS; and increased LV wall thickness and impaired basal LS.
