# Supplementary material for: Electrocardiographic Characteristics and Their Correlation with Echocardiographic Alterations in Fabry Disease
Source: J Cardiovasc Dev Dis. 2022 Jan 3;9(1):11. doi: 10.3390/jcdd9010011 (PMC8777656; doi:10.3390/jcdd9010011)
Supplement: Supplementary file 1 [file jcdd-09-00011-s001.zip › jcdd-1491926-supplementary.pdf]

Table S1. Electrocardiographic characteristics of FD patients in comparison to echocardiographic findings. ECG parameters assessed are listed in the first column. Comparison is made between FD patients with increased LV wall thickness and normal LV wall thickness; FD patients with impaired GLS vs normal GLS; and between FD with impaired basal LS and normal basal LS.

	**FD Patients with Increased vs. Normal LV Wall Thickness**			**FD Patients with Impaired GLS (Worse Than -18.0%) Compared to Normal GLS**			**FD Patients with Impaired Basal LS (Worse Than -18.1%) Compared to Normal Basal LS**		
	**Increased LV Wall Thickness [n=27]**	**Normal LV Wall Thickness [n=18]**	**p-Value**	**Impaired GLS [n=28]**	**Normal GLS [n=16]**	**p-Value**	**Impaired Basal LS [n=34]**	**Normal Basal LS [n=10]**	**p-Value**
Age (yrs)	44	38	0.060	47	35	**0.001 †**	45	34	**0.007 †**
Corrected PQ Interval (ms)	156	141	0.328	157	141	0.327	156	133	0.249
Corrected PWD (ms)	96	85	0.328	94	89	0.960	95	82	0.356
QRS duration (ms)	101	88	**0.044 †**	99	93	0.700	98	90	0.578
R wave amplitude lead I (mV)	9.9	5.4	**0.002 †**	9.7	5.7	**0.008 †**	9.1	5.1	**0.014 †**
Sokolow-Lyon index (mV)	27.7	21.8	0.112 †	27.5	21.8	0.125	26.5	21.4	0.235
Sokolow-Lyon LVH criteria	13/27 (48%)	1/18 (6%)	**0.003 †**	12/28 (43%)	2/16 (13%)	**0.049 †**	14/34 (41%)	0/10	**0.018 †**
Modified Cornell Index (mV)	5.4	2.3	**0.016 †**	4.9	3.2	0.076	4.8	2.2	0.133
Modified Cornell LVH Criteria	4/27 (15%)	0/18	0.138	3/28 (11%)	1/16 (6%)	0.620	4/34 (12%)	0/10	0.559
RBBB criteria	4/27 (15%)	1/18 (6%)	0.634	4/28 (14%)	1/16 (6%)	0.638	5/34 (15%)	0/10	0.573

Intervals and voltage are expressed as Mean. Categorical variables are expressed as frequency and percentage of occurrence in each group. **†***p < 0.05*. PWD = P-wave duration; LVH = Left Ventricular Hypertrophy; RBBB = Right bundle branch block; GLS = Global longitudinal strain; LS = longitudinal strain.

Table S2. Baseline clinical and echocardiographic characteristics of FD patients.

Age (yrs)	42
ERT – no. on ERT at time of simultaneous ECG / TTE	7 (16%)
ERT – total no. who were treated with ERT after ECG / TTE	26 (58%)
Albuminuria / Chronic kidney disease	13 (29%)
Dyslipidaemia	21 (47%)
Diabetes mellitus	3 (7%)
Arterial hypertension	17 (38%)
Transient ischaemic attack / stroke	8 (18%)
Ischaemic heart disease	3 (7%)
Echocardiographic parameters	
Septal wall thickness (mm)	12.2
Posterior wall thickness (mm)	11.3
Average LV wall thickness (mm)	11.7

Left ventricular mass index (g/m^2)	113.6
LVEF (%)	61
Peak E (cm/sec)	85
Peak A (cm/sec)	65
E/A	1.40
Lateral e′ (cm/sec)	10
Septal e′ (cm/sec)	8
Average E/e′	10.1
LAVI (mL/m^2)	38.7

Table S3. Comparison of clinical, echocardiographic and electrocardiographic markers of FD patients with normal LV wall thickness (cut off 13mm). and normal basal LS; normal LV wall thickness with impaired basal LS; and increased LV wall thickness and impaired basal LS.

	**Normal LV Wall Thickness (<13mm) & Normal Basal LS [n=10] [Group A]**	**Normal LV Wall Thickness (<13mm) & Impaired Basal LS [n=20] [Group B]**	**Increased LV Wall Thickness (>13mm) & Impaired Basal LS [n=14] [Group C]**
**Clinical characteristics**			
Age	34	41	**51 +**
Males	3 (30%)	13 (65%)	10 (71%)
ERT – no. on ERT at time of simultaneous ECG/TTE	0	4 (20%)	3 (21%)
ERT – total no. who were treated with ERT after ECG/TTE	2 (20%)	12 (60%)	12 (86%)
Albuminuria / Chronic kidney disease	0	5 (25%)	8 (57%)
Dyslipidaemia	2 (20%)	9 (45%)	10 (71%)
Diabetes mellitus	0	1 (5%)	2 (14%)
Arterial hypertension	2 (20%)	8 (40%)	9 (64%)
Transient ischaemic attack / stroke	1 (10%)	4 (20%)	3 (21%)
Ischaemic heart disease	1 (10%)	1 (5%)	2 (14%)
**Echocardiographic parameters**			
Average LV wall thickness (mm)	9.1	10.1	**16.5 +**
Left ventricular mass index (g/m^2)	83.8	104.6	**153.5 +**
LVEF (%)	61	62	60
Peak E (cm/sec)	94	86	76
Peak A (cm/sec)	64	63	69
E/A	1.56	1.51	1.14
Lateral e′ (cm/sec)	13	11	**8 +**
Septal e′ (cm/sec)	10	**9 ***	**7 +**
Average E/e′	8.82	9.78	**11.9 +**
LAVI (mL/m^2)	38	42	37
Valvular disease	Moderate MR – 1<br>Mitral prolapse – 1 (mild)<br>Mild TR – 1	Moderate MR – 2<br>Moderate PR – 1<br>Mild MR - 2<br>Mild TR – 4<br>Mild AS – 1	Moderate MR – 1<br>Mild MR – 1<br>Mild TR – 1
**ECG paramaters**			
Corrected PQ Interval (ms)	133	150	164
Corrected PWD (ms)	86	92	99
QRS duration (ms)	90	95	104
Amplitude lead I (mV)	5.1	8.6 *	10.0
Sokolow-Lyon index (mV)	21.4	25.7	27.8

Sokolow-Lyon LVH criteria	0	8 (40%)	6 (43%)
Modified Cornell index (mV)	2.2	4.3	5.6
Modified Cornell LVH criteria	0	2 (10%)	2 (14%)
RBBB criteria	0	3 (15%)	2 (14%)

Intervals and voltage are expressed as Means. Categorical variables are expressed as frequency and percentage of occurrence in each group. †$p<0.05$. PWD = P-wave duration; LVH = Left Ventricular Hypertrophy; RBBB = Right bundle branch block; GLS = Global longitudinal strain; LS = longitudinal strain; LVEF = Left ventricular ejection fraction; LAVI = Left atrial volume index; MR = mitral regurgitation; PR = pulmonary regurgitation; TR – tricuspid regurgitation; AS = aortic stenosis. * $p < 0.05$ for Group A vs Group B. + $p < 0.05$ for Group B vs. Group C.
